# Supplementary figures and images for: Comprehensive analysis of the correlations of S100B with hypoxia response and immune infiltration in hepatocellular carcinoma
Source: PeerJ. 2022 Mar 29;10:e13201. doi: 10.7717/peerj.13201 (PMC8973469; doi:10.7717/peerj.13201)

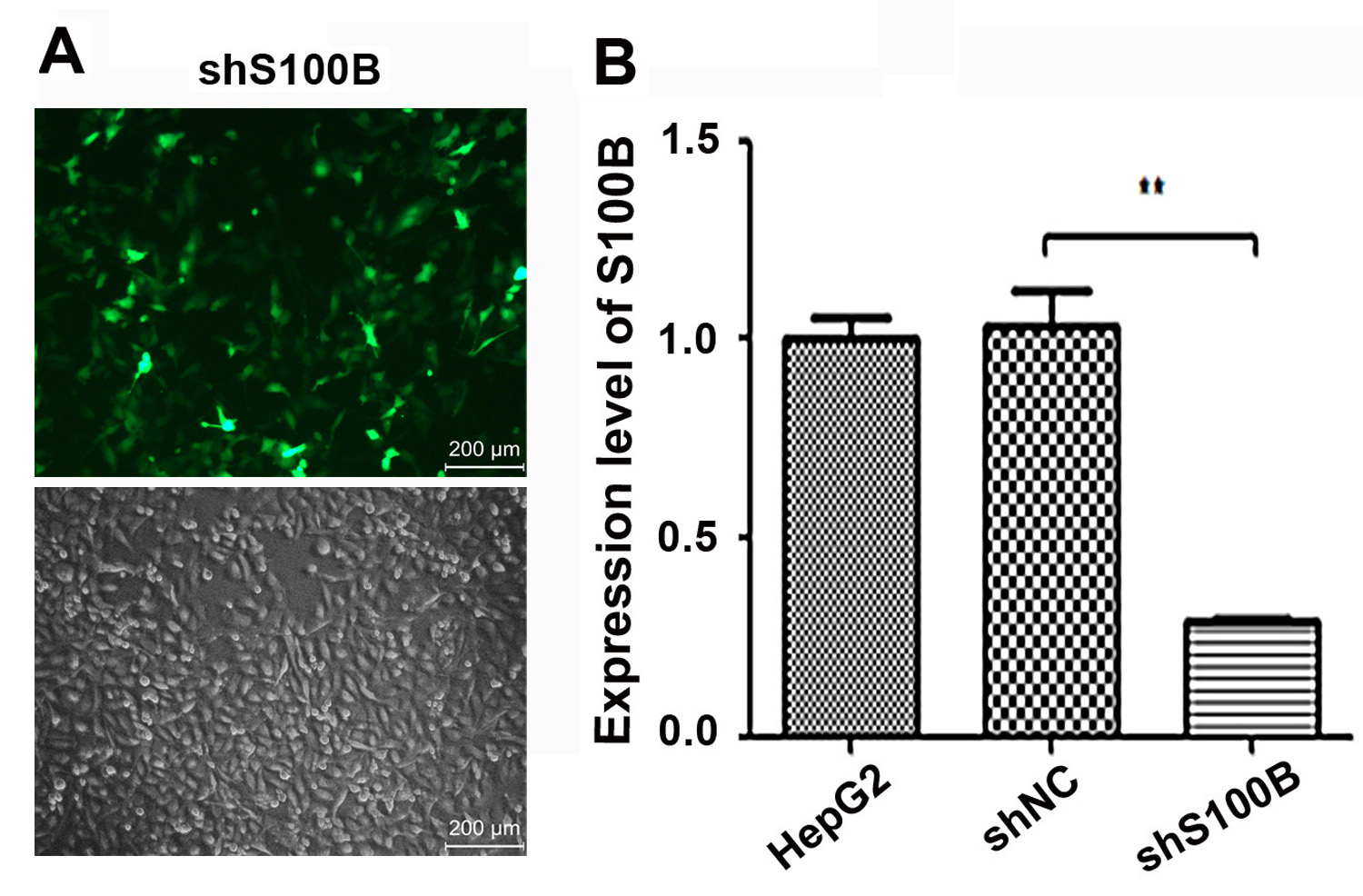

Supplement: Supplemental Information 1 — (A, B) The shRNA targets S100B is transfected into HepG2 cells to downregulated S100B expression. (A) The fluorescence of cells after shRNA transfection in HepG2 cells. bar = 200 μm. (B) The mRNA level of S100B in shRNA transfected HepG2 cells. Data are shown as the mean ± standard deviation based on three independent experiments. **P < 0.01. [file peerj-10-13201-s001.png]

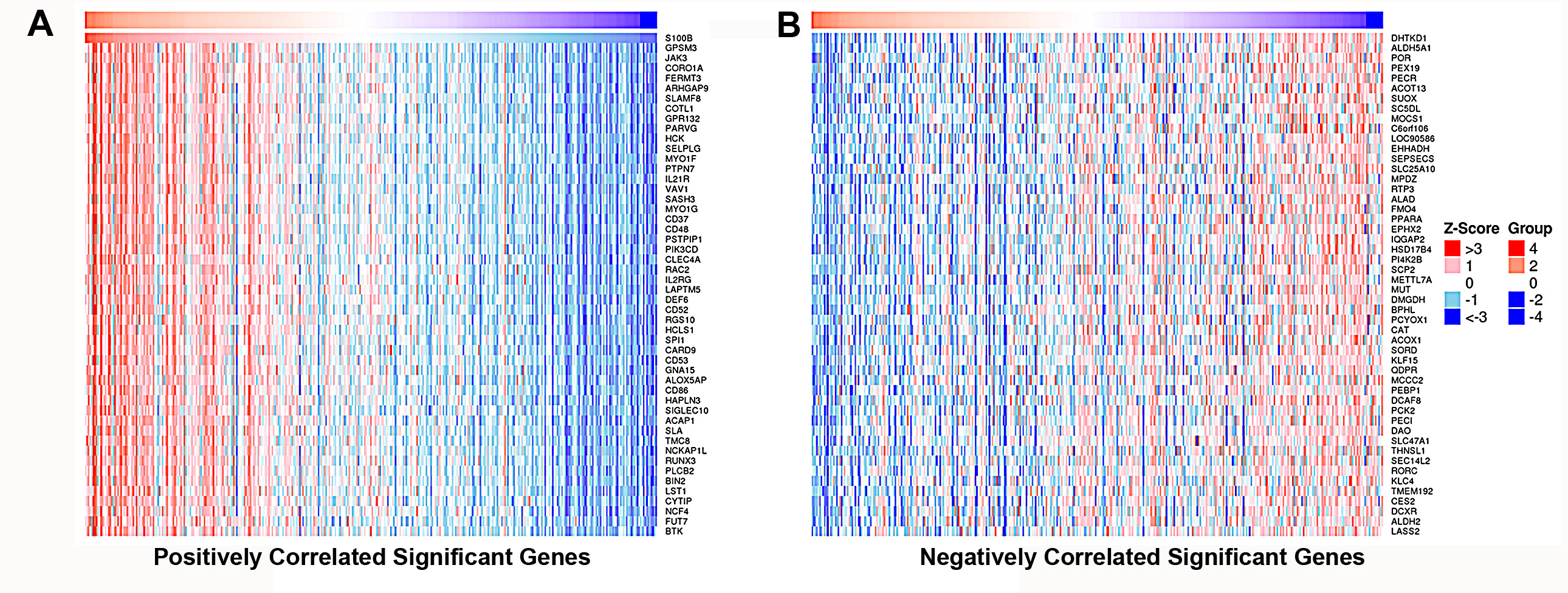

Supplement: Supplemental Information 2 — (A, B) Heatmaps of the top 50 genes positively and negatively correlated with S100B, respectively. Orange represents positive correlation; blue represents negative correlation. [file peerj-10-13201-s002.png]

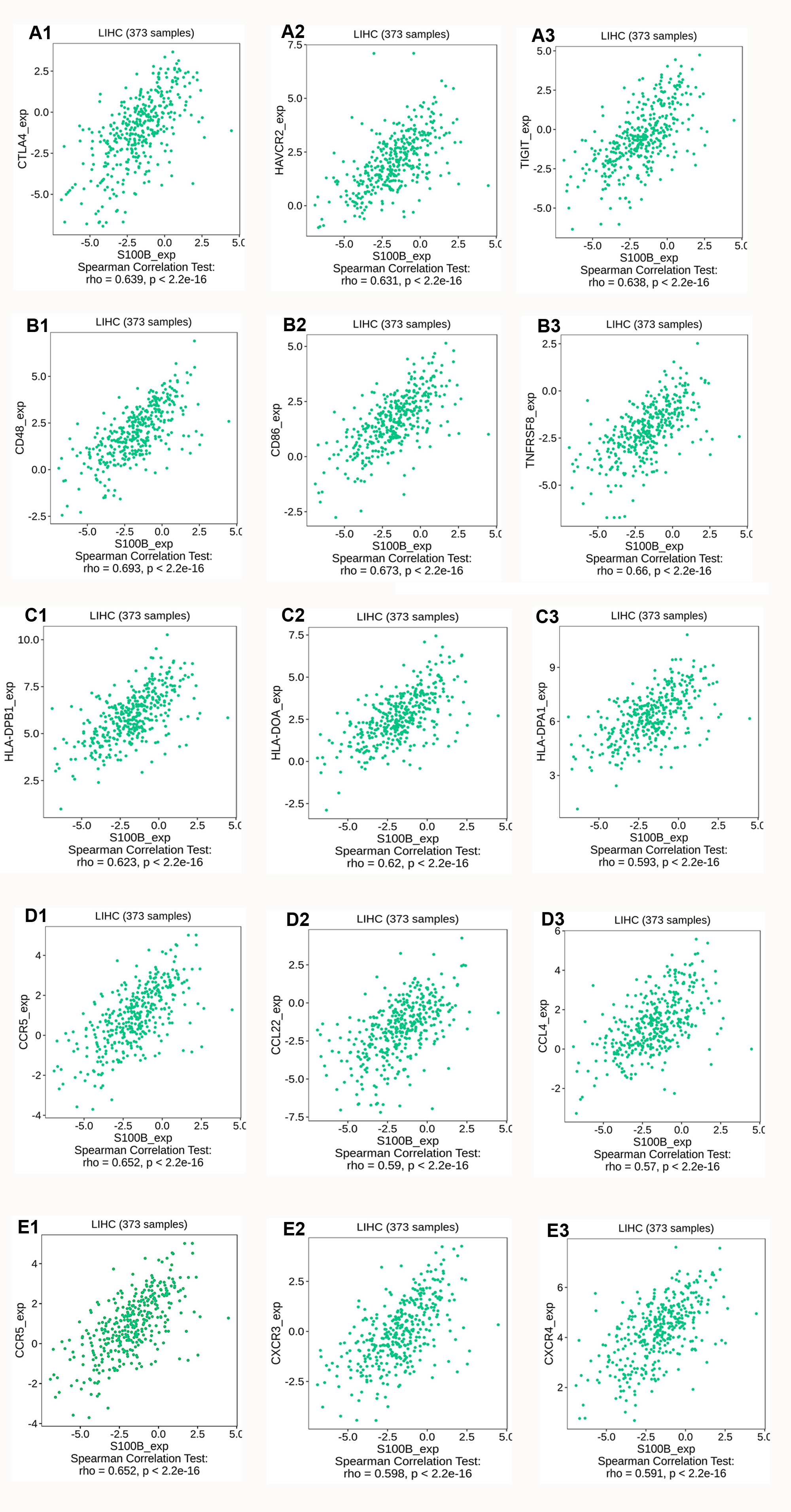

Supplement: Supplemental Information 3 — The top three immune-inhibitors (A) and immune-stimulators (B) associated with S100B expression in HCC. (C) The top three MHCs correlated with S100B expression in HCC. (D) The top three chemokines and (E) chemokine receptors involved in S100B expression in HCC. [file peerj-10-13201-s003.png]
